# Cytosolic Malic Enzyme 1 (ME1) Mediates High Fat Diet-Induced Adiposity, Endocrine Profile, and Gastrointestinal Tract Proliferation-Associated Biomarkers in Male Mice

**DOI:** 10.1371/journal.pone.0046716

**Published:** 2012-10-04

**Authors:** Ahmed Al-Dwairi, John Mark P. Pabona, Rosalia C. M. Simmen, Frank A. Simmen

**Affiliations:** 1 Department of Physiology and Biophysics, University of Arkansas for Medical Sciences, Little Rock, Arkansas, United States of America; 2 Winthrop P. Rockefeller Cancer Institute, University of Arkansas for Medical Sciences, Little Rock, Arkansas, United States of America; 3 Arkansas Children’s Nutrition Center, University of Arkansas for Medical Sciences, Little Rock, Arkansas, United States of America; National Cancer Institute, United States of America

## Abstract

**Background:**

Obesity and associated hormonal disturbances are risk factors for colon cancer. Cytosolic Malic Enzyme (ME1) generates NADPH used for lipogenesis in gastrointestinal (GI), liver and adipose tissues. We have reported that inclusion of soy protein isolate (SPI) in the diet lowered body fat content and colon tumor incidence of rats fed AIN-93G diet, while others have demonstrated SPI inhibition of rat hepatic ME1 expression. The present study examined the individual and combined effects of dietary SPI and absence of ME1 on: 1) serum concentrations of hormones implicated in colon cancer development, 2) expression of lipogenic and proliferation-associated genes in the mouse colon and small intestine, and 3) liver and adipose expression of lipogenic and adipocytokine genes that may contribute to colon cancer predisposition.

**Methods:**

Weanling wild type (WT) and ME1 null (MOD-1) male mice were fed high-fat (HF), iso-caloric diets containing either casein (CAS) or SPI as sole protein source for 5 wks. Somatic growth, serum hormone and glucose levels, liver and adipose tissue weights, GI tissue parameters, and gene expression were evaluated.

**Results:**

The MOD-1 genotype and SPI-HF diet resulted in decreases in: body and retroperitoneal fat weights, serum insulin, serum leptin, leptin/adiponectin ratio, adipocyte size, colon mTOR and cyclin D1 mRNA abundance, and jejunum FASN mRNA abundance, when compared to WT mice fed CAS-HF. Regardless of diet, MOD-1 mice had reductions in liver weight, liver steatosis, and colon crypt depth, and increases in adipose tissue expression of IRS1 and IRS2, compared to WT mice. SPI-HF diet reduced ME1 gene expression only in retroperitoneal fat.

**Conclusions:**

Data suggest that the pharmacological targeting of ME1 or the inclusion of soy protein in the diet may provide avenues to reduce obesity and its associated pro-tumorigenic endocrine environment and improve insulin sensitivity, potentially disrupting the obesity-colon cancer connection.

## Introduction

The prevalence of overweight and obesity has escalated during the last several decades [1]. Obesity is a major risk factor for the development of diabetes, insulin resistance, metabolic syndrome, cardiovascular diseases, and several cancers, including those of the colon [2]. Chronic consumption of high fat-containing diets is a contributor to both obesity and colon cancer [3], [4]. Further, obesity and colon cancer are frequently associated with systemic hormonal changes, particularly with high serum levels of insulin and leptin [5], two hormones that are thought to promote cancer initiation and progression [3], [6], [7].

Insulin-induced lipogenesis is a known survival mechanism by which colorectal cancer cells maintain growth, escape apoptosis and acquire chemo-resistance [8]–[11]. This pathway is known to be regulated by dietary and hormonal factors [12]. Up-regulation of insulin signaling in liver and adipose tissues increases steatosis and fat mass, respectively and the same pathway supports growth and chemo-resistance of cancer cells [13]. In this regard, pharmacologic inhibition or knockdown of Fatty Acid Synthase (FASN) and Acetyl-CoA Carboxylase (ACC), key enzymes of lipid synthesis in rapidly proliferating cells, can lead to cancer cell growth arrest and apoptosis [8], [14], [15].

Adipose tissues secrete a number of adipocytokines to regulate food intake, energy balance and fat deposition [16]; these include leptin and adiponectin. Chronic consumption of fat leads to increased adipose tissue mass, leptin secretion and leptin resistance, resulting in hyperleptinemia [16]. Leptin stimulates proliferation of colon cancer cell lines *in vitro* [6], [7]. On the other hand, adiponectin which is negatively correlated with body fat mass, also plays a major role in energy balance and insulin sensitization [16], and may inhibit colon tumor cell proliferation [17]. Accumulating evidence suggests that low serum adiponectin levels constitute a risk factor for colon cancer development [18], [19]. A high leptin/adiponectin ratio has been correlated with worse metabolic and pathologic outcomes of obesity [20], [21].

Cytosolic malic enzyme (ME1) catalyzes the reversible oxidative decarboxylation of malate to pyruvate, carbon dioxide and NADPH, the latter contributing to *de novo* fatty acid synthesis by FASN [22]. Mice express two ME1 protein variants, due to differential RNA splicing, which share ∼95% amino acid sequence [23]. A mutation in the mouse ME1 gene that eliminated ME1 protein functionality was first reported by Lee *et al* in 1980 [24]; these mice were designated MOD-1. MOD-1 mice appear normal and reproduce [24]. Recent studies with mice and humans have linked liver- and adipose-expressed ME1 with pre-disposition to obesity and type 2 diabetes [25], [26]. However, the influence of ME1 on the endocrine profile and its impact on colon and intestinal cell proliferation remain unknown.

The type of dietary protein consumed may affect metabolic outcome [27]. Consumption of soy protein and soy isoflavones is generally associated with health benefits, including decreased adiposity and colon cancer risk [8], [28]–[30]. Here, we examined effects of soy protein diet and ME1 null mutation on serum concentrations of hormones implicated in colon cancer risk. Using wild type (WT) and MOD-1 mice, we determined the effects of iso-caloric HF diets formulated with casein (CAS) as a control protein, or soy protein isolate (SPI) on body weight, adiposity, serum concentrations of insulin and adipocytokines, liver and adipose tissue morphology, and expression of lipogenic, proliferation-associated, and adipocytokine genes. Our data identify roles for both ME1 and dietary protein type in mediating murine endocrine profile, body composition and adipose, liver and GI gene expression and suggest that attenuated ME1 expression/function may provide increased protection from colon cancer.

## Methods

### Experimental Animals and Diets

**Figure 1:**
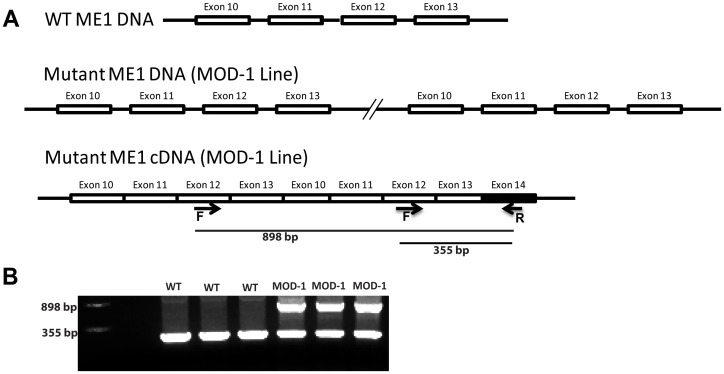
Schematic representation of the ME1 gene segment duplication in the MOD-1 mouse line. (A), Locations of PCR primers (F: forward and R: reverse) located in exons 12 and 14. A region encompassing exons 10, 11, 12, and 13 is duplicated in tandem within this locus in the MOD-1 line. (B), RT-PCR of ME1 RNA transcripts in WT and MOD-1 mouse jejunum. MOD-1 tissues produced two products of different size due to the segment duplication.

All animal experiments were carried out under protocols approved by the University of Arkansas for Medical Sciences Institutional Animal Care and Use Committee. Iso-caloric diets contained a high level of fat (45%) and as sole protein sources, either casein (CAS) or soy protein isolate (SPI). Diets were formulated by Harlan Laboratories (Madison, WI). Solae, LLC (St. Louis, MO) provided the SPI and corresponding isoflavone analysis for the batch of SPI used. Diet composition is described in Table 1. Wild type (WT) and MOD-1 breeders (both lines in C57BL/6N background) were obtained from Merck & Co., Inc. (Whitehouse Station, NJ), courtesy of Taconic Laboratories (Germantown, NY). Animals were housed in the animal facility at the University of Arkansas for Medical Sciences under a 12 h light/12 h dark cycle. Mice were maintained as homozygous WT or MOD-1 lines. WT and MOD-1 female mice were fed AIN-93G diet (Harlan) prior to breeding and during pregnancy and lactation. Male progeny were weaned at postnatal day (PND) 21 to one of the two experimental diets (10 mice per genotype/diet group). Mice were weighed weekly and *ad libitum* fed CAS-HF or SPI-HF diet for 5 wks (until PND 56) at which time blood, intestines, and liver and gonadal and retroperitoneal adipose tissues were obtained. Small intestine was divided into three equal lengths; the middle third region was considered the jejunum. Colon was divided into two equal lengths. The distal half was used for protein and gene expression analyses, while the distal ∼5 mm of the proximal half (designated mid-colon) was fixed and embedded in paraffin for immunohistochemistry and histological analyses. Genotypes were confirmed by Western blot of ME1 in liver and intestine, and by RT-PCR of ME1 RNA in jejunum [primers located in exons 12 and 14 (forward: 5′-AATTCCTACGTGTTCCCTGG-3′, and reverse: 5′-ATCCTAGCTGTTGCGTTACTG-3′) (Fig. 1)].

**Table 1:** Composition of study diets.

Component	CAS-HF (g/Kg)	SPI-HF(g/Kg)
Isolated Soy Protein*	0	210
Casein	210	0
L-Cystine	3	1.2
L-Methionine	0	2.5
Corn Starch	160	160
Maltodextrin	150	150
Sucrose	150	150
Lard	210	210
Corn Oil	20	20
Cellulose	28.96	28.26
Mineral Mix	50	50
Vitamin Mix	15	15
Choline Bitartrate	3	3
TBHQ, antioxidant	0.04	0.04

Measurements are g of ingredient per Kg of diet. CAS-HF: Casein-High Fat diet, SPI-HF: Soy Protein Isolate-High Fat diet. Each diet provided 45% of total calories from fat and 4.7 Kcal/g of energy.

* Isoflavone content of the SPI-HF diet was 246 mg genistein and 167 mg daidzein per Kg diet.

### RNA Isolation and Quantitative Real Time, Reverse Transcriptase - polymerase Chain Reaction (qRT-PCR)

RNA was extracted from individual distal colons and jejunums, livers and retroperitoneal fat depots (n = 5–7 animals/group/tissue) with TRIZol reagent (Life Technologies, Grand Island, NY). One ug of total RNA was reverse-transcribed to cDNA using the iScript cDNA synthesis kit (Bio-Rad; Hercules, CA). Expression of target genes was assayed by qRT-PCR using Bio-Rad iTaq SYBR Green Supermix. Primers used in qRT-PCR (Table 2) were obtained from Integrated DNA Technologies, Inc. (Coralville, IA). The primers used to quantify ME1 mRNA were designed to amplify both murine ME1 RNA splice variants. Target mRNA abundance in distal colon was normalized to a factor derived from the geometric mean of expression values for 18S rRNA, β-actin, cyclophilin A and TATA box binding protein (TBP), calculated using GeNorm [31]. Target mRNA abundance in jejunum was normalized to a factor derived from the geometric mean of expression values for β-actin, GAPDH and TBP. Target mRNA abundance in liver and fat was normalized to a factor derived from the geometric mean of expression values for β-actin, cyclophilin A and TBP. Two-way ANOVA and Student’s *t* test were used to compare variables between groups (SigmaPlot 11; Systat Software, Inc.; Chicago, IL).

**Table 2:** List of primers used in RT-PCR.

Gene	Forward Primer (5′–3′)	Reverse Primer (5′–3′)
β-Actin	CCACAGGATTCCATACCCAAG	ACCGTGAAAAGATGACCCAG
Cyclophilin A	GCAGACAAAGTTCCAAAGACAG	CATTATGGCGTGTAAAGTCACC
GAPDH	CTTTGTCAAGCTCATTTCCTGG	TCTTGCTCAGTGTCCTTGC
18S rRNA	GAGACTCTGGCATGCTAACTAG	GGACATCTAAGGGCATCACAG
TBP	AAGAAAGGGAGAATCATGGACC	GAGTAAGTCCTGTGCCGTAAG
ME1	AGTATCCATGACAAAGGGCAC	ATCCCATTACAGCCAAGGTC
FASN	CCCCTCTGTTAATTGGCTCC	TTGTGGAAGTGCAGGTTAGG
Leptin	AGCCTCACTCTACTCCACAG	CCTCTACATGATTCTTGGGAGC
Adiponectin	TGTCTGTACGATTGTCAGTGG	GCAGGATTAAGAGGAACAGGAG
IRS1	ATAGCGTAACTGGACATCACAG	GCATCGTACCATCTACTGAAGAG
IRS2	GTCCAGGCACTGGAGCTTT	GCTGGTAGCGCTTCACTCTT
Ki67	TGCCCGACCCTACAAAATG	GAGCCTGTATCACTCATCTGC
Cyclin D1	GCCCTCCGTATCTTACTTCAAG	GCGGTCCAGGTAGTTCATG
mTOR	ATTCAATCCATAGCCCCGTC	TGCATCACTCGTTCATCCTG

### Protein Isolation and Western Blot Analysis

Tissues were homogenized in RIPA buffer containing a protease inhibitor cocktail (Santa Cruz Biotechnology; Santa Cruz, CA) and protein concentrations were determined using the BCA (bicinchoninic acid) protein assay kit (Pierce; Rockford, IL). Proteins (70 μg) were separated in 10% SDS-PAGE gels and transferred to a nitrocellulose membrane (Bio-Rad). Membranes were incubated overnight with primary antibodies raised against ME1 (1/1000 dilution; Abcam, Cambridge, MA) and β-actin (1∶10,000 dilution; Sigma Aldrich, St. Louis, MO). Antibodies were diluted in Odyssey blocking buffer (Li-Cor Biosciences; Lincoln, NE). Membranes were placed in blocking buffer for 1 h, washed and then incubated with appropriate horseradish peroxidase-conjugated secondary antibody for 1 h. Protein bands were visualized using chemiluminescence (Amersham Bioscience; Piscataway, NJ). Densitometry of x-ray films was performed with Alpha View software (Cell Biosciences; Santa Clara, CA). Levels of ME1 were normalized to that of β-Actin and two-way ANOVA was used to examine for significant differences between groups.

### Serum Hormone Concentrations

Animals were euthanized in the fed state between 8–11 a.m. and trunk blood was collected and centrifuged to obtain serum. Serum insulin, leptin and adiponectin concentrations [8–10 animals/group (PND 56)] were measured using Milliplex MAP Mouse Adipokine kits (Millipore Corp., Billerica, MA). Serum IGF-I concentration was measured by mouse/rat IGF-I ELISA (R&D Systems, Inc.; Minneapolis, MN). Each individual mouse serum was assayed in duplicate.

### Histology and Immunohistochemistry

Mid-colon tissue was fixed in 10% neutral-buffered formalin (pH 7.4) for overnight, and embedded in paraffin. Five µm sections were stained with hematoxylin and eosin (H&E). For immunohistochemistry, paraffin-embedded mid-colon samples were serially sectioned, dewaxed, and rehydrated through a graded alcohol series as previously described [32]. Antigen unmasking was performed by boiling the sections using Citra Plus (Biogenex, San Ramon, CA) in a microwave oven for 2 min at power 10 and then for 10 min at power 1, followed by cooling for 20 min. Sections were treated with 3% hydrogen peroxide to quench endogenous peroxidase activity and incubated in blocking solution containing goat IgG (Vectastain Elite ABC kit, Vector Laboratories, Inc.; Burlingame, CA, USA) for 30 min. Sections were incubated overnight with rabbit ME1 polyclonal antibody (Proteintech Group, Inc.; Chicago, Illinois) followed by incubation with goat anti-rabbit secondary antibody (Vectastain Elite ABC kit; Vector Laboratories) for 30 min. Sections were stained with 3,3′-diaminobenzidine tetra-hydrochloride (Dako Inc.; Carpinteria, CA) and counterstained with hematoxylin. Slides were dehydrated in an alcohol series and cleared in xylene. Pictures were acquired and analyzed with Aperio ImageScope (Aperio Technologies Inc.; Vista, CA). Colon crypt depths were measured and an average for 6–10 crypts per animal (with 5–6 animals examined per group) was determined.

Liver tissues taken for analysis of lipid content were frozen in OCT compound, cut at 5 μm thickness, stained with Oil Red O, and counterstained with hematoxylin. Pictures were acquired and analyzed with Aperio ImageScope. Lipid droplet staining intensity was determined using Aperio software and relative staining was determined, using 3 animals per group.

Retroperitoneal fat tissue was snap-frozen in liquid nitrogen and stored at −80°C. For histological analysis, fat tissues were embedded in OCT compound and snap-frozen in isopentane, previously chilled in liquid nitrogen; 5 µm thick sections were cut and stained with hematoxylin and eosin (H&E). Pictures were acquired and analyzed with Aperio ImageScope. Individual adipocyte cell area was measured and an average determined for 150–200 cells per animal (3 animals/group).

## Results

### MOD-1 Mouse Line

MOD-1 mice have an internally duplicated segment within the ME1 gene that encompasses exons 10–13 (Fig. 1A). This internal duplication results in formation of an aberrant ME1 mRNA encoding an unstable ME1 protein variant that does not accumulate to appreciable levels in cytoplasm [33]. We verified expression of the aberrant mRNA in MOD-1 mice and its absence in WT mice (Fig. 1B).

### Weight Gain and Fat Deposition

**Figure 2:**
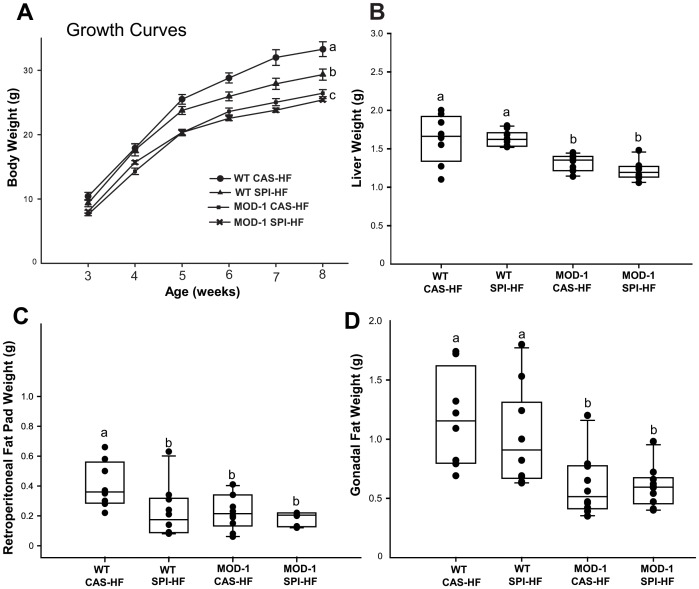
Growth of WT and MOD-1 mice fed the experimental diets. (A), Data points of body weights are mean ± SEM. Box plots of final tissue weights for: (B), liver; (C), retroperitoneal fat; and (D), gonadal fat. Boxes indicate the inter-quartile range of 25–75% and median; dots represent individual animals (n = 8–10/group). Lowercase letters (a, b, c) indicate significant differences (P < 0.05).

Male WT and MOD-1 mice were weaned to HF diet containing either casein or SPI (Table 1, Fig. 2A). Weaning weights did not differ between the assigned groups. WT and MOD-1 mice exhibited their most rapid weight gains during the first 2 weeks (age 3 to 5 weeks) of consuming the experimental diets (Fig. 2A). Although food consumption did not differ among groups (data not shown), MOD-1 mice gained significantly less weight than did WT mice. During weeks 5–8, SPI-HF-fed WT mice showed significantly reduced weight gains compared to WT counterparts fed CAS-HF diet; MOD-1 mice had less weight gains than corresponding WT mice, regardless of diet (Fig. 2A). At the end of the experimental period, body weights were greater for WT mice fed the CAS-HF diet (mean: 33.25 ± 0.71 g) than for WT mice fed SPI-HF (mean: 29.32 ± 0.7 g, P<0.001) and both MOD-1 groups (means; CAS-HF: 26.42 ± 0.71 g, SPI-HF: 25.37 ± 0.71 g, P<0.001).

We examined effects of dietary protein and ME1 genotype on liver and fat tissue weights. MOD-1 mice had reduced liver (by ∼20%) and gonadal fat (by ∼50%) tissue weights when compared to corresponding tissues from WT mice; however, no dietary effects on these indices were observed (Figs. 2B, D). By contrast, MOD-1 mice fed either diet or WT mice fed SPI-HF diet had significantly reduced weight of the retroperitoneal fat depot (by ∼45%) when compared to WT mice fed with the CAS-HF diet (Fig. 2C).

### Serum Insulin, Leptin, Adiponectin and IGF-I Concentrations

**Figure 3:**
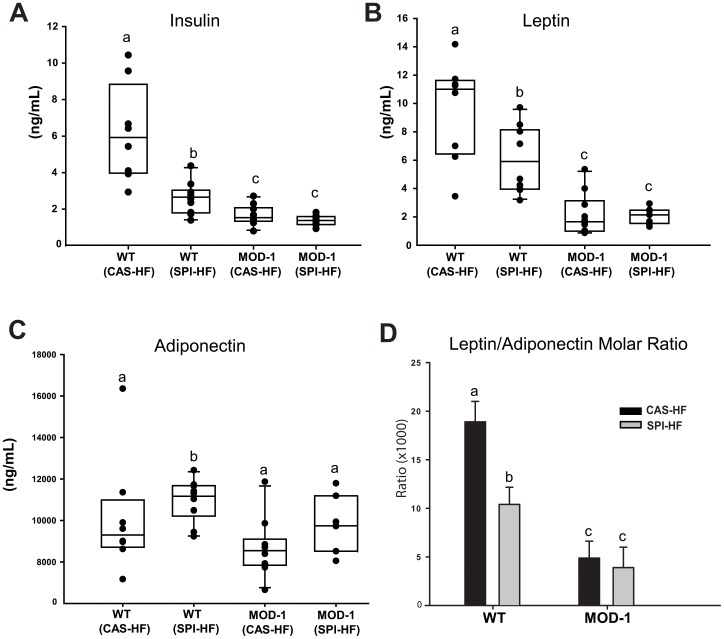
Serum hormone concentrations at study termination. Box plots for: (A), insulin; (B), leptin; and (C), adiponectin concentrations. Boxes indicate the inter-quartile range of 25–75% and median; dots represent individual animals (n = 8–10/group). (D), leptin/adiponectin molar ratio (mean ± SEM). Lowercase letters (a, b, c) indicate significant differences (P < 0.05).

In WT mice, serum insulin concentrations were reduced by ∼50% with SPI-HF diet when compared to CAS-HF diet (Fig. 3A). MOD-1 mice fed either diet had further reductions in insulin concentrations (by ∼70%) compared to CAS-HF-fed WT mice (Fig. 3A). Random blood glucose concentrations did not differ between any of the treatment groups (data not shown). Serum leptin concentrations were reduced by ∼40% in WT mice fed SPI-HF and were further attenuated in MOD-1 mice fed either diet when compared to CAS-HF-fed WT mice (Fig. 3B). Adiponectin concentrations in the WT mice were elevated with the SPI-HF diet, but did not differ in MOD-1 mice fed either diet (Fig. 3C). Leptin/adiponectin molar ratio was significantly reduced by ∼45% in WT mice fed SPI-HF and by ∼75% in MOD-1 mice fed either diet when compared to WT mice fed CAS-HF (Fig. 3D). In contrast, serum IGF-I concentrations did not differ among the groups (data not shown).

### Effects of Diet and ME1 Genotype on Gene Expression in Distal Colon and Jejunum

**Figure 4:**
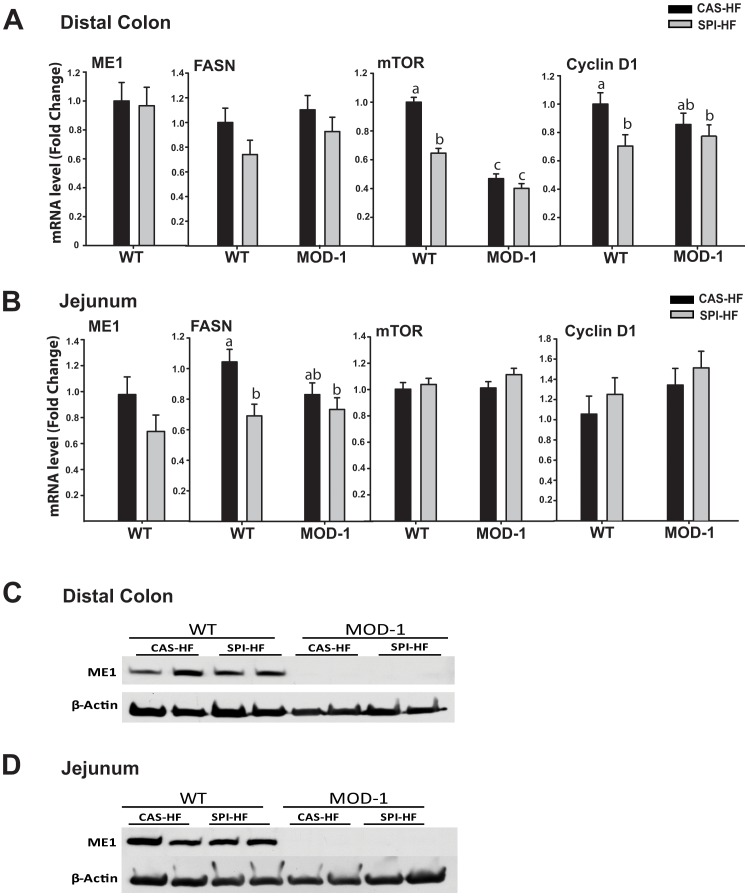
Relative expression of mRNAs encoding the lipogenic enzymes ME1 and FASN and pro-proliferative proteins mTOR and cyclin D1. (A), Distal colons and (B), jejunums of WT and MOD-1 mice of the two diet groups. Data are mean ± SEM of fold change in normalized expression (n = 5–7 mice/group) relative to WT mice fed the CAS-HF diet. Lowercase letters (a, b, c) indicate significant differences (P < 0.05). (C, D), Western blots of ME1 in the distal colons and jejunums from individual animals (n = 4/group) show absence of ME1 in MOD-1 mice, and lack of dietary effect on ME1 protein levels in WT mice.

To evaluate potential consequences of altered endocrine profiles with ME1 null genotype and dietary protein, we examined the intestinal expression of lipogenic enzyme genes ME1 and Fatty Acid Synthase (FASN) and of the mammalian target of rapamycin (mTOR), a downstream mediator of insulin signaling. Real-time quantitative RT-PCR was performed on RNA preparations from distal colons (DC) and jejunums of individual mice. Jejunum was included since this constitutes a major site of fat and nutrient absorption and thus, may affect adiposity, endocrine profile and propensity for colon cancer development. ME1 mRNA abundance was unaffected by diet type in both the distal colon and jejunum of WT mice (Fig. 4A, B). Absence of ME1 (MOD-1 mice) resulted in significantly decreased transcript levels of colon mTOR and jejunum FASN, relative to WT mice fed CAS-HF diet (Fig. 4A, B). SPI-HF-fed mice tended to recapitulate the effects of ME1 absence on colon mTOR and jejunum FASN expression, when compared to WT counterparts (Fig. 4A, B). Expression analysis of the cyclin D1 gene as a marker of proliferation indicated comparable effects of ME1 absence and dietary SPI in distal colon, but no significant effects in the jejunum (Fig. 4A, B). Relative mRNA abundance for Ki67, another proliferation-associated protein, was reduced in the jejunum with SPI-HF and with MOD-1 genotype, but was unaffected by diet or treatment in the distal colon (data not shown). Western blots confirmed the absence of ME1 protein in MOD-1 distal colon and jejunum; moreover, there were no dietary effects on levels of ME1 protein in WT mice (Figs. 4C, D).

### Effects of Diet and ME1 Genotype on Colon Crypt Depth

**Figure 5:**
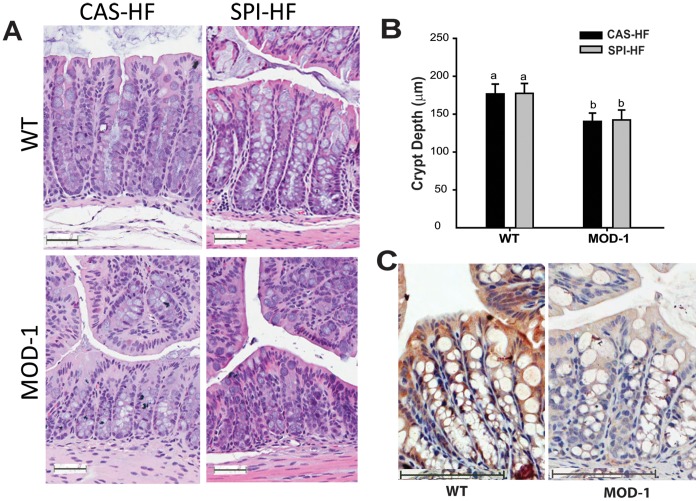
Effects of dietary SPI and ME1 genotype on proliferative status of the colon. (A), Representative images of H&E-stained sections of mid-colons from WT and MOD-1 mice fed CAS-HF or SPI-HF diets. Scale bars: 50 µm. (B), Analysis of crypt depth from 5–7 mice/group; 6–10 crypts were measured for each animal. Data represent the mean ± SEM. Means with different lowercase letters differed at P < 0.05. Different lowercase letters (a, b) indicate significant differences for P < 0.05. (C), Immunohistochemistry of ME1 in mid-colon of WT and MOD-1 mice, demonstrating cytoplasmic localization in crypt epithelium and luminal epithelium of WT mice. Representative images are shown. Scale bars: 100 µm.

We next examined for effects of SPI-HF diet and/or absence of ME1 on colon crypt depth, a surrogate biomarker for colon crypt stem/progenitor cell activity. MOD-1 mice had significantly shallower crypts (mean: 141.3 ± 8.5 µm) than WT mice (mean: 177.1 ± 9.28) (P<0.01); however, no dietary effects on colon crypt depth were observed with either genotype (Figs 5A, B). Immunohistochemical staining for ME1 protein demonstrated its cytoplasmic localization in WT mouse colon crypt epithelium and luminal epithelium, and absence in MOD-1 mouse colon (Fig. 5C).

### Effects of Diet and ME1 Genotype on Hepatic Gene Expression and Hepatosteatosis

**Figure 6:**
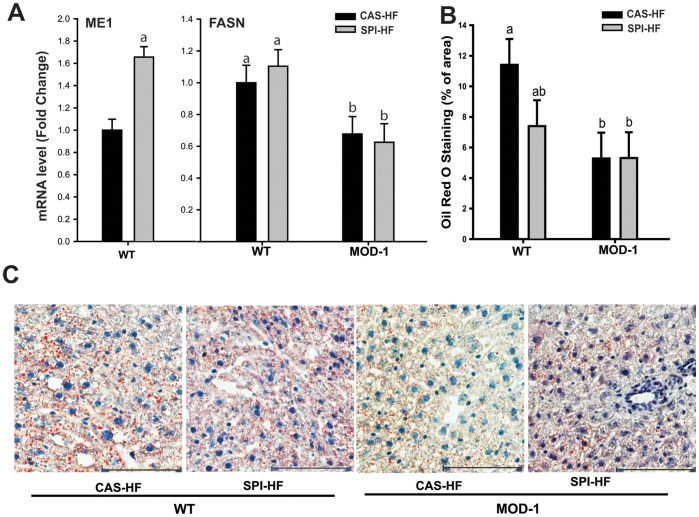
Hepatic lipogenic enzyme gene expression and lipid content. (A), Relative expression levels of mRNAs encoding ME1 and FASN in the livers of WT and MOD-1 mice of the two diet groups. Data are mean ± SEM of fold change in normalized expression (n = 7 mice/group), relative to WT mice fed CAS-HF diet. (B), Lipid droplet quantity after Oil Red O staining of livers from WT and MOD-1 mice of the two diet groups (n = 3 mice/group). Lowercase letters (a, b) indicate significant differences (P < 0.05). (C), Representative images of liver tissues stained with Oil Red O, scale bar: 100 µm.

To determine the effects of SPI-HF diet and ME1 null mutation on liver lipogenic state, we examined hepatic ME1 and FASN gene expression and lipid content, the latter by staining with Oil Red O. Interestingly, SPI-HF diet induced ME1 expression in WT mice without altering FASN mRNA expression (Fig. 6A). However, FASN mRNA expression was reduced in MOD-1 mouse liver, regardless of diet (Fig. 6A). Hepatosteatosis, as gauged by Oil Red O staining, was significantly reduced in MOD-1 mice independent of diet type (Fig. 6B, C) and consistent with the FASN gene expression data. However, SPI-HF diet elicited a decrease in liver steatosis in WT mouse liver (Fig. 6B, C).

### Effects of Diet and ME1 Genotype on Adipose Gene Expression and Morphology

**Figure 7:**
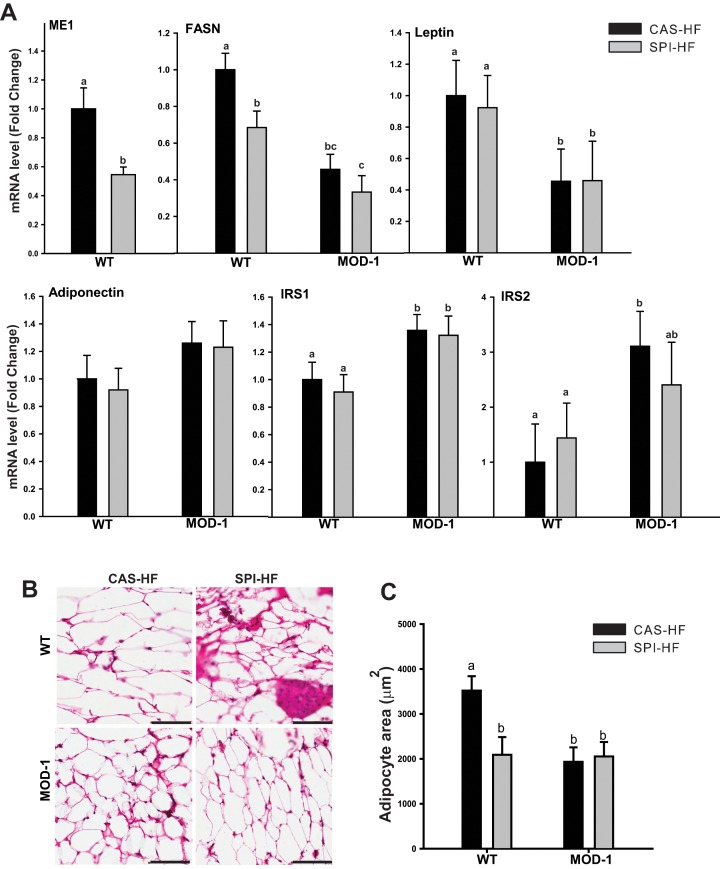
Effects of diet and genotype on adipose tissue gene expression and adipocyte cell size. (A), Relative expression of mRNAs encoding lipogenic enzyme genes (ME1, FASN), adipocytokines (leptin, adiponectin), and insulin receptor substrates (IRS1 and IRS2) in the retroperitoneal adipose tissue from WT and MOD-1 mice of the two diet groups. Data are mean ± SEM of fold change in normalized expression (n = 5 mice/group), relative to WT mice fed CAS-HF. (B), Representative images of H&E stained adipose tissue. (C), Quantification of adipocyte cell size measured using Aperio software (n = 3 mice/group). Scale bars: 100 µm. Lowercase letters (a, b, c) indicate significant differences (P < 0.05).

Data presented above showed that retroperitoneal fat mass was reduced by SPI-HF diet as well as by ME1 null mutation. Thus, we sought to determine effects of diet and MOD-1 genotype on fat cell gene expression and size. Fig. 7A depicts relative expression of mRNAs encoding ME1 and FASN, the adipocytokines leptin and adiponectin, and the insulin receptor signal transducers, IRS1 and IRS2, in the retroperitoneal fat depot. SPI-HF diet decreased expression of ME1 and FASN in WT mice, likely indicating a reduced state of lipogenesis. FASN expression was reduced in fat of MOD-1 mice, regardless of the diet consumed. Interestingly, MOD-1 retroperitoneal fat manifested decreased expression of leptin, consistent with the serum leptin data, and increased expression of IRS1 and IRS2, with no accompanying changes in adiponectin expression. Histological examination of fat tissue morphology (Fig. 7B) revealed a significant reduction (∼50%) in adipocyte size in MOD-1 mice comparable with those of SPI-HF-fed WT mice. Results are consistent with reduced lipogenic gene expression and suppressed fat storage as a consequence of dietary SPI or MOD-1 genotype.

## Discussion

Obesity is a major risk factor for colon cancer development in rodents and humans [34], [35]. Chronic high fat consumption is a primary cause of obesity and insulin resistance; however, dietary proteins and carbohydrates also have an impact on resultant metabolic and endocrine consequences and thus, potentially on colon cancer risk [12], [27]. Previous work using rodent models found inhibitory effects of dietary SPI on: a) colon and mammary cancers, b) hepatosteatosis, c) adipose tissue deposition, and d) mammary and liver ME1 expression [28], [36]–[38]. These published data suggested a linkage between metabolism, adiposity and tumorigenic status, possibly involving ME1. The present study is the first to evaluate the individual and combined effects of anti-obesogenic soy protein isolate intake and ME1 deficiency on systemic and tissue parameters that are known to affect propensity for colon cancer development. A particular focus was on whether ME1 mediates any of the effects of SPI.

We used male mice for our studies since the obesity-colon cancer relationship (i.e., relative risk) in humans is stronger for men than women [34]. Our results showed that the ME1 gene as well as SPI diet, significantly impact the endocrine profile of mice when challenged with obesogenic diet, and which potentially affects colon cancer propensity. Both consumption of SPI and the absence of ME1 protein (MOD-1 mice) conferred protection against development of high fat diet-induced adiposity and its attendant putative pro-tumorigenic endocrine environment. This was reflected in the observed reductions in body weights, liver and fat tissue weights, serum insulin and leptin concentrations, leptin/adiponectin ratios, hepatosteatosis, and white adipose cell size, relative to WT-CAS-HF-fed mice. These data extend previous research on the anti-obesogenic effects of soy-based diets in rodent models [30], [36], [39] and the MOD-1 mouse phenotype [25], [26].

Systemic hormonal factors are presumed to be important for tumor development by virtue of their induction/repression of genes and proteins associated with cell survival, proliferation, apoptosis and autophagy [7], [17], [40], [41]. Our analysis of FASN, ME1, mTOR, Cyclin D1 and Ki67 gene expression revealed significant changes associated with dietary protein type and presence or absence of ME1, but within specific tissue contexts. These responses manifested regional variations (colon *vs.* jejunum) which most likely reflected differential responses to the local and systemic environments, as well as differences in specific tissue architecture and cell physiology. mTOR is an important signal transducer downstream of the PI3K/AKT pathway that mediates the effects of multiple growth factors, is up-regulated and highly active in multiple cancers including those of the colon, and is a drug target for cancer therapeutics [42], [43]. FASN is robustly expressed in many solid tumors and early tumorigenic lesions in the colon and supports tumor cell proliferation and survival through enhanced lipogenesis and anti-apoptosis [44], [45]. Given that FASN and mTOR are mediators of insulin action [46], [47] and ME1 has been proposed to augment insulin secretion [48], the decrease in proliferation-associated gene expression with dietary SPI and loss of ME1 is consistent with attenuated insulin action in distal colon and jejunum. The observed reduction in colon mTOR gene expression with dietary SPI also may also be related to levels of leptin, adiponectin and/or other serum factors; alternatively, it may reflect inhibition by as yet unknown bioactive component(s) in SPI [49], [50]. With regard to the above, leptin is known to induce/activate mTOR via the PI3K/AKT pathway [51], whereas adiponectin has been shown to suppress this gene through the AMPK pathway [17]. Soy isoflavones, at high levels, can induce apoptosis and growth arrest of cells through direct inhibition of mTOR in certain cancer cell lines [49]. Taken together, results suggest that the endocrine status associated with dietary SPI and/or ME1 null genotype exerts considerable influence on the expression of key proliferation and lipogenic genes in the colon and small intestine.

**Figure 8:**
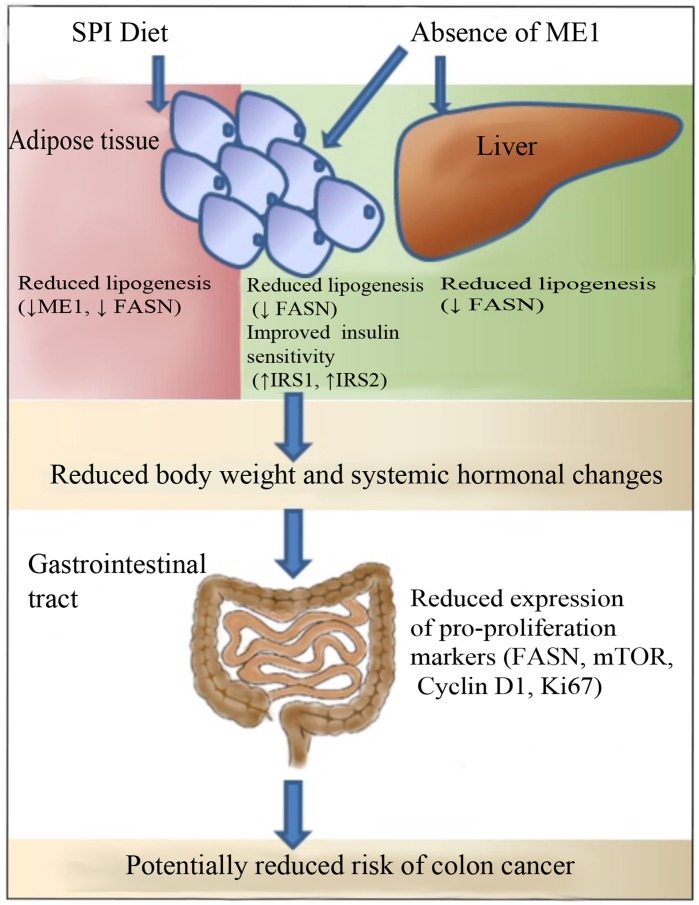
A proposed integrative model for how dietary soy protein and ME1 null mutation may affect colon cancer risk/propensity. The targeting of adipose tissue (SPI diet, ME1) and liver (ME1) results in reduced body weight and systemic hormonal changes that favor reduced colon cancer risk. The expression of genes influenced by dietary SPI or ME1 null mutation is summarized here for each tissue.

Serum insulin and its more stable surrogate biomarker, C-peptide, are significant risk factors for colon cancer in both men and women [52]. Exogenous insulin increased tumor incidence and tumor multiplicity in rats given the intestinal carcinogen azoxymethane [53]. In a previous study from our laboratory, lifetime consumption of SPI lowered: tumor incidence, body weight accretion and serum insulin and leptin levels in a non-obese, rat model of colon cancer [38]. Serum leptin is a significant risk factor for human colon cancer [54] and leptin enhanced colon tumor cell proliferation in obese mouse models [7]. Adiponectin, in contrast, is a suppressor of intestinal tumorigenesis and intestinal epithelial cell proliferation in rat and mouse models [19], [55], [56]. A high leptin:adiponectin ratio has been correlated with increased risk for developing insulin resistance in obese and non-obese individuals [20], [57], [58]. The decrease in leptin:adiponectin ratios in MOD-1 and SPI-HF-fed WT mice, compared to CAS-HF- fed WT mice, further substantiates the linkage between the cancer-preventive effects of SPI consumption and reduction in ME1 expression. However, dietary SPI did not mimic the effects of loss of ME1 on all serum and tissue parameters we evaluated in MOD-1 mice. For example, we did not observe reductions in ME1 expression in jejunum and distal colon of WT mice fed HF-SPI, relative to WT mice fed CAS-HF. Rather, SPI caused a reduction in ME1 expression only in retroperitoneal fat, indicating a tissue-specific effect of SPI. This reduced ME1 expression was correlated with lower FASN expression and adipocyte size, consistent with a previous publication [37]. Similarly, absence of ME1 in MOD-1 mice conferred a state of reduced lipogenesis in fat, as inferred from diminished FASN expression and cell size. Interestingly, SPI induced ME1 expression in livers of WT mice, similar to what we previously reported in genetically obese rats [59]. However, this induction was not associated with increased FASN gene expression or hepatosteatosis, which suggests that the anti-steatotic effect of SPI is via another pathway(s) (such as fatty acid oxidation) or gene. Nonetheless, MOD-1 mice had significant reductions in lipogenic enzyme gene expression and steatosis in the liver, suggesting direct or indirect ME1 action. In contrast, colon ME1 is likely not a direct target of SPI (and its associated endocrine milieu); rather, SPI may act indirectly, most probably through adipose-expressed ME1 to influence adiposity and growth and tumorigenic potential of the distal colon (model in Fig. 8).

MOD-1 mice fed either diet had shallower crypts than WT mice. This points to a direct or indirect stimulatory effect of ME1 on colon stem-progenitor cell proliferation, although these possibilities remain speculative at present. Recently, O’Brien et al. reported that the Drosophila intestine adapts to feeding conditions, by altered intestinal stem cell dynamics as a consequence of perturbations in gastrointestinal insulin levels [60]. Overfeeding increased local insulin production, which led to a significant increase in gut dimensions, whereas fasting reduced insulin levels and gut dimensions. Although MOD-1 mice had a marked reduction in serum insulin levels, this can only partially explain the differences in crypt depth, since SPI, which significantly lowered serum insulin levels in WT mice, had no effect on colon crypt depth. Given the higher IRS1 and IRS2 mRNA expression in white adipose tissue of MOD-1 mice, indicative of increased insulin sensitivity [61], in MOD-1 mice, but not in that of WT mice fed SPI-HF diet, our data suggest that enhanced insulin sensitivity may lead to reduced steady-state insulin levels and subsequently, attenuated proliferative state within the GI tract with loss of ME1.

### Conclusions

In summary, the absence of functional ME1 protein affects body weight, adiposity, endocrine profile and colon crypt depth of male mice fed a high fat diet. Dietary soy protein consumption elicited favorable physiological effects including reduced body weights and retroperitoneal adiposity. Both the absence of ME1 as well as SPI consumption led to reduced lipogenic gene expression in retroperitoneal adipose tissue and which undoubtedly contributed to the changes in adiposity. We speculate that colon and jejunum are tissue targets of these favorable effects. However, many of the associations observed here are correlative and thus, require experimental confirmation. In this regard, future studies using tissue-specific null alleles of ME1 may provide a way to confirm or refute various aspects of the integrative model presented in Fig. 8. The collective data suggest that dietary intervention by soy-based diet, and/or pharmacologic targeting of ME1 to prevent excessive adipose tissue deposition and improve endocrine parameters and insulin sensitivity also may provide protection against colonic epithelial cell transformation. The inability of dietary SPI to recapitulate all of the effects of ME1 null mutation may reflect in part, the total absence of ME1 in MOD-1 mice (extreme case) as opposed to the reduced ME1 expression in SPI-HF diet-fed WT animals. Our observations that SPI did not further enhance effects of MOD-1 mutation on physiologic indices, is consistent with ME1 as a major component in the SPI-HF diet response.
